# Diaspora contributions: evidence from the Outliers network building in Guatemala

**DOI:** 10.3389/frma.2026.1852444

**Published:** 2026-08-25

**Authors:** Camila Sáenz, Cindy García, Paulina Briz, María Klose, Andrea Carrascosa, Francis Sanchinelli

**Affiliations:** 1 College of Management, National Taipei University of Technology, Taipei, Taiwan; 2 Independent Researcher, Guatemala City, Guatemala

**Keywords:** community, diaspora engagement, Guatemala networking, innovation ecosystems, Outliers, professional networks

## Abstract

This community case study examined Outliers, a network-based community launched in 2024 to reconnect members of Guatemala's professional and scientific diaspora with local entrepreneurs and foreign residents committed to contributing positive change in the country. The study documented outcomes and lessons from the community's first year, focusing on strengthening national innovation capacity through structured networking and collaborative exchange. Using a mixed-methods case study design, data were drawn from survey responses of 93 members and follow-up qualitative insights to explore participants' motivations, engagement patterns, perceived value creation, and barriers to sustained involvement. Findings revealed high cross-sector diversity, strong demand for meaningful professional connections, and evidence of trust-building and knowledge exchange across borders. Members reported forming new collaborations and accessing expertise otherwise unavailable within local networks. Key limitations included time constraints, coordination challenges, and the community's early-stage governance structure.

This research contributes to the literature on diaspora knowledge networks by providing empirical evidence that intentionally designed professional communities can facilitate early-stage relational infrastructure in emerging innovation ecosystems. It underscores how structured engagement of diaspora actors can strengthen national innovation capacity, promote cross-border collaboration, and support sustainable development initiatives in resource-constrained contexts.

Outliers addresses these gaps by mobilizing dispersed professional and scientific networks to enhance persistent challenges related to access to specialized talent, fragmented innovation ecosystems, and the underutilization of knowledge and expertise embedded within its professional and scientific diaspora, collective problem-solving, and innovation. The case highlights the potential of intentional network design to bridge skills gaps and facilitate knowledge transfer in emerging economies.

## 1 Introduction

Guatemala, located in the heart of Central America, is the region's largest economy, with a Gross Domestic Product (GDP) exceeding $110 billion as of 2025 (International Monetary Fund, 2025). Over the past decade, the country has maintained relative macroeconomic stability, characterized by an average annual growth rate of approximately 4%, underpinned by prudent fiscal and monetary policies (International Trade Administration, 2026). Despite these favorable macroeconomic indicators, Guatemala continues to face structural challenges that constrain long-term development, including persistent institutional weaknesses coupled with chronically low public spending and underinvestment in critical sectors such as health, education, and infrastructure. These limitations are particularly consequential given Guatemala's demographic profile. With a population of approximately 18 million (International Monetary Fund, 2025) and more than 60% of whom are of working age, the country possesses substantial human capital. However, recent national employment data indicate that nearly 70% of the workforce operates within the informal sector, where productivity levels and opportunities for professional advancement are scarce (Instituto Nacional de Estadística, 2025). As a result, a widening gap has emerged between the capabilities of the workforce and the opportunities available domestically to deploy those capabilities effectively. In this context, socioeconomic mobility has become a central pathway for international migration. This dynamic increasingly extends beyond low-skilled workers to include students and highly educated professionals seeking academic, institutional, and labor environments better suited to the effective application of their skills. This pattern is not unique to Guatemala, but rather reflects a broader trend observed across Latin America. As a result, Guatemala experiences a sustained outflow of human capital, particularly toward countries in the Global North, where higher wages, stronger professional networks, and career trajectories that remain largely unavailable at home are more accessible (Bonilla et al., 2022a,b). Estimates suggest that over 1.5 million Guatemalans reside abroad, primarily in the United States of America (Migration Policy Institute, 2022; International Organization for Migration, n.d.). Public discussion around migration has traditionally emphasized its financial dimension, as remittances are equivalent to more than 19% of the country's GDP (International Organization for Migration, n.d.) and constitute a critical source of household income and macroeconomic stability.

While undeniably important, this emphasis often overlooks another key dimension of migration, the transfer of knowledge, social capital, and professional networks. Although highly skilled Guatemalans do not constitute the largest proportion of the country's migrant population, their relevance remains significant due to the advanced academic training, professional expertise, and strategic institutional positions they often occupy abroad. A growing segment of Guatemalan migrants consists of educated professionals who have developed academic and professional expertise in international contexts (Bonilla et al., 2022a,b), carrying with them valuable human capital, institutional knowledge, and access to global innovation systems. This experience creates an opportunity to strengthen connections between globally distributed talent and Guatemala's professional ecosystem (Alvarado et al., 2022; Barrios-Guzmán and de la Cruz, 2022). As the country experiences growing entrepreneurial activity and gradual strengthening of its innovation system, evidence from the Global Entrepreneurship Monitor (2023) suggests that progress depends not only on investment but also on effective collaboration among universities, private sector actors, entrepreneurs, and public institutions. In this context, fostering connections across geographic boundaries becomes essential to enhancing knowledge exchange, collaboration, and overall innovation capacity (Meyer and Brown, 1999a,b; Tejada, 2012).

Founded in 2024, Outliers is a network-based community designed to connect members of Guatemala's professional and scientific diaspora with locally embedded entrepreneurs, and foreign residents committed to the country's development. The community seeks to strengthen Guatemala's emerging innovation ecosystem through intentional relationship-building, cross-sector collaboration, and knowledge exchange across geographic boundaries. By creating recurring spaces for interaction among globally connected and locally engaged professionals, Outliers aims to facilitate trust formation, collaborative problem-solving, and the circulation of expertise that may otherwise remain fragmented across disconnected networks, consistent with literature emphasizing the developmental role of diaspora knowledge networks and transnational professional communities (Meyer and Brown, 1999a,b; Brinkerhoff, 2008).

## 2 Brain gain and diaspora knowledge networks: rationale for outliers

The emigration of highly skilled and educated individuals from developing countries to advanced economies in pursuit of greater professional opportunities, commonly known as “brain drain” (Bonilla et al., 2022a,b; Docquier and Rapoport, 2012), remains a significant challenge for many emerging economies seeking to strengthen their competitiveness, productivity, and long-term economic growth. Numerous studies have suggested that persistent outflow of professionals may weaken the research, innovation, and development capacities of their countries of origin, particularly where scientific and technological systems face institutional or financial constraints (Docquier et al., 2007). However, contemporary research increasingly challenges the brain drain of highly skilled individuals necessarily constitutes a permanent loss of knowledge and talent. During the final decades of the 20th century, groups of highly skilled individuals that emigrated from developing countries to advanced economies began to organize and establish connections both among themselves and with their countries of origin. These transnational networks gave rise to what literature has variously termed *intellectual diaspora networks, scientific, technological and economic diasporas*, and more recently, *diaspora knowledge networks* (DKNs) (Meyer and Wattiaux, 2006).

Building on this perspective, DKNs emphasize the potential of globally dispersed professionals to contribute to the development of their countries of origin through knowledge transfer, collaboration, mentorship, and the creation of institutional linkages across geographic contexts (Meyer and Brown, 1999a,b; Tung, 2008; Tejada, 2012). Under this perspective, diaspora professionals can be understood not as lost human capital, but as reservoirs of distributed knowledge that can support innovation and collective learning when connected through appropriate structures that encourage interaction and collaboration.

Since the 1990s, the academic debate on diaspora engagement has evolved considerably, moving from skepticism regarding the practical viability of such networks to recognition of their tangible development impact as empirical evidence accumulated. A pivotal contribution came from Meyer and Wattiaux (2006), whose systematic review of DKNs reframed the traditional brain drain narrative, into one of brain gain through skills circulation, in which emigrants' expertise becomes a remotely deployable, yet accessible resource embedded in transnational networks. Despite their diversity, DKNs share a common objective: supporting development in their countries of origin by mobilizing members' knowledge, experience, and professional capacities back home. In doing so, these networks have fundamentally reshaped how highly skilled mobility is understood, shifting the focus from one-way talent loss to the strategic mobilization of globally dispersed professionals for national development.

Building on this foundation, Brinkerhoff (2008) offered a comprehensive framework for understanding the full scope of diaspora contributions to development. Moving beyond the traditional focus on financial remittances, Brinkerhoff argued that diaspora communities generate at least three interrelated forms of capital: financial, human, and social. While monetary transfers are the most visible component, the human capital embodied in professional expertise, technical skills, and tacit knowledge acquired abroad, together with the social capital embedded in trust-based networks and institutional linkages, may hold far greater transformative potential. When these resources are mobilized effectively, they can strengthen innovation systems, facilitate technology transfer, and expand access to international markets.

Crucially, however, such outcomes rarely emerge spontaneously. The developmental impact of diasporas depends not only on the existence of structured mechanisms that facilitate sustained interaction between globally dispersed professionals and locally embedded actors, but also on the ability to properly identify, document, and mobilize diaspora resources in ways that generate measurable economic and social value. As a result, diaspora engagement becomes less about reversing brain drain and more about transforming dispersed talent into a coordinated problem-solving network. Intentional network design therefore plays a central role in this process, creating spaces where relationships can form, information can circulate, and collaborative initiatives can emerge across geographic boundaries.

Outliers represents one such effort in Guatemala. Founded in 2024, the community was designed to connect members of Guatemala's professional and scientific diaspora with local entrepreneurs and foreign residents committed to the country's development. Drawing on the principles of diaspora knowledge networks, the community seeks to transform globally dispersed professionals into a resource for collaboration, knowledge transfer, and collective problem-solving. Through structured networking, recurring gatherings, and peer-to-peer interactions, Outliers fosters connections among individuals who might not otherwise engage within Guatemala's fragmented innovation ecosystem. During its first year of operation, the community brought together more than 200 members from diverse professional backgrounds and geographic locations, creating opportunities for sustained exchange and collaboration across sectors and borders.

This case study examined how an intentionally designed professional community could mobilize diaspora talent, facilitate knowledge transfer, and contribute to the development of relational infrastructure, the networks of trust, professional ties, and recurring spaces for interaction that enable collaboration, information exchange, and collective action within an emerging innovation system. Drawing on survey data collected during the community's first operational phase, from its launch in September 2024 through December 2025, the study analyzed participant characteristics, motivations for engagement, interaction patterns, and perceived value creation. In doing so, it explored both the opportunities and the practical challenges associated with leveraging DKNs as mechanisms for collaboration, innovation and national development.

As a community, Outliers highlights the importance of relational infrastructure consistent with literature on diaspora knowledge networks (Brinkerhoff, 2008; Meyer and Brown, 1999a,b). In this study, relational infrastructure refers specifically to the social connections, trust relationships, and recurring interaction spaces, documented through open-ended survey responses-including peer introductions, informal exchanges at events, and cross-sector dialogue.

This interpretation aligns with literature suggesting that sustained interaction spaces and trust-based connections are important in enabling distributed human capital to develop into collaborative problem-solving capacity (Brinkerhoff, 2008; Meyer and Brown, 1999a,b). While traditional innovation strategies often focus on institutions, funding, or formal programs such as government-sponsored science networks or international cooperation initiatives, this study suggests that trust-building among individuals can also contribute to knowledge exchange and the circulation of ideas across sectors and geographies, consistent with literature on diaspora networks and transnational knowledge exchange (Bonilla et al., 2022a,b; Meyer and Wattiaux, 2006).

## 3 Methodological approach

The study employed a mixed methods research design integrating quantitative descriptive analysis with qualitative thematic interpretation to examine the structural, behavioral, and relational dynamics of the Outliers community in Guatemala. Mixed-methods approaches are particularly well-suited to community studies where numerical patterns alone are insufficient to capture the motivational and relational complexity of human networks (Creswell and Plano Clark, 2017; Tashakkori and Teddlie, 2010).

### 3.1 Research design

The study drew on a cross-sectional survey design as its primary instrument of data collection. Cross-sectional designs allow for the simultaneous capture of multiple variables across a population at a single point in time, enabling both descriptive profiling and exploratory correlational analysis (Bryman, 2016). The survey instrument: the Outliers community intake form, was structured to capture information across six dimensions: (i) professional background and industry expertise; (ii) entrepreneurial experience and venture participation; (iii) technical and strategic capabilities; (iv) willingness to collaborate and contribute to the ecosystem; (v) geographic location and professional networks; and (vi) intended engagement within the community. The instrument combined closed ended items used for quantitative profiling with a set of open-ended fields capturing qualitative reflections on returnee motivations, perceived barriers to engagement, and perceived roles for the community; on average, completion required approximately 5 min.

### 3.2 Sampling strategy

The study adopted a non-probability purposive sampling approach, whereby participants were intentionally selected based on characteristics and experiences that are particularly relevant to the research objectives (Patton, 2002; Miles et al., 2014). The sample consisted of 93 confirmed members and applicants of the Outliers Community. This approach is particularly appropriate because Outliers itself operates as a community with a defined membership selection process. To join the Outliers Guatemala community, applicants submit a request for consideration, which is reviewed and approved by the Outliers core team. Candidates are assessed based on their entrepreneurial profile, national and international experience, commitment to the country, and willingness to engage in collaborative knowledge exchange.

### 3.3 Data collection

Survey responses were collected digitally through an online form distributed directly by email to all community members with a 3-week deadline established for the submission of completed responses ensuring accessibility for the community's internationally distributed membership. Participation was voluntary, and respondents were informed that their data would be used for research and community development purposes. All data were anonymized before analysis in accordance with standard ethical guidelines for social research (American Psychological Association, 2020; Economic and Social Research Council, 2015).

### 3.4 Analytical approach

Quantitative analysis followed a two-stage procedure. In the first stage, frequency distributions and descriptive statistics were computed to characterize the demographic and professional composition of the community, including age, gender, sector representation, years of international experience, entrepreneurial status, and event attendance. This approach followed established practice in community network research (Wasserman and Faust, 1994). In the second stage, cross-tabulations and pattern analysis were used to explore the relationships between variables such as motivational profiles, collaboration intent, trust formation, and perceived barriers to engagement. These exploratory comparisons allowed the identification of structural patterns within the community, including the gap between relational intent and behavioral activation, consistent with frameworks developed in diaspora knowledge network scholarship (Meyer and Brown, 1999a,b; Brinkerhoff, 2008). Qualitative analysis was applied to open-ended survey fields, including responses on returnee motivations, barriers to engagement, and perceived roles for the community, using inductive thematic analysis as outlined by Braun and Clarke (2006). Coding proceeded in two stages: initial line-by-line open coding generated descriptive labels, followed by axial coding in which related labels were grouped into four higher-order thematic categories through iterative comparison and discussion among the research team, enabling interpretive depth beyond what closed-item quantitative data alone can yield. The integration of quantitative and qualitative findings followed a convergent parallel design (Creswell and Plano Clark, 2017), wherein both strands are analyzed independently and then synthesized at the interpretive stage to produce a richer, more complete account of community dynamics.

## 4 Findings and discussion

### 4.1 Community profile

Survey results indicated that the Outliers community is composed primarily of early and midcareer professionals, with 66% of respondents between the ages of 25 and 34. In addition, 62% of respondents identified as female. This female-majority participation is notable given that, despite comparatively high levels of female entrepreneurship in Latin America and the Caribbean, persistent gender gaps remain. Previous regional research based on GEM data found that male early-stage entrepreneurial activity exceeded female activity in all the Latin American and Caribbean economies examined except Peru (Avolio Alecchi, 2020). Moreover, although almost 50% of private firms in the region have at least one woman among their owners, women account for only 14% of board members and 4% of CEOs in listed companies (de Hoop et al., 2024). The result therefore suggests particularly strong female engagement among respondents in the Outliers community, rather than representing the gender composition of the broader regional entrepreneurial population. This demographic profile of the Outliers community, rather than that of the broader Guatemalan population, suggests a community positioned at a stage of professional development often characterized by active network building, skill accumulation, and the exploration of new career and entrepreneurial opportunities (Lévesque and Minniti, 2006).

A defining characteristic of the Outliers community is its strongly transnational profile combined with sustained ties to Guatemala. Survey responses indicated that 88% of participants have lived outside Guatemala, with 65% residing abroad for 3 years or more, nearly 60% for more than 5 years, and over a quarter for longer than a decade. Despite this extensive international exposure, respondents reported enduring connections to Guatemala: 82% hold Guatemalan nationality and an additional 12% possess dual citizenship. Furthermore, 52% identified themselves as professionals primarily based abroad, 35% as individuals bridging local and international contexts, and 14% as professionals primarily based in Guatemala. This combination of international mobility and local attachment reflected what Levitt and Glick Schiller (2004) describe as dual embeddedness, whereby individuals maintain meaningful connections to multiple social and national contexts simultaneously. Rather than functioning as a traditional diaspora association, understood here as a formally registered, membership based organization, Outliers operates as an informal, curated professional community, bringing together professional and scientific diaspora members, local entrepreneurs, and foreign residents committed to Guatemala's development, and creating a space where globally informed perspectives could interact with locally grounded knowledge. Consistent with this interpretation, 36% of respondents who belonged to other professional networks identified access to global perspectives, understood here as exposure to international professional norms, market practices, and cross border opportunities not readily available through domestic networks, as a key reason for joining the community, underscoring Outliers's role as a bridge between local and international professional spheres.

**Figure 1:**
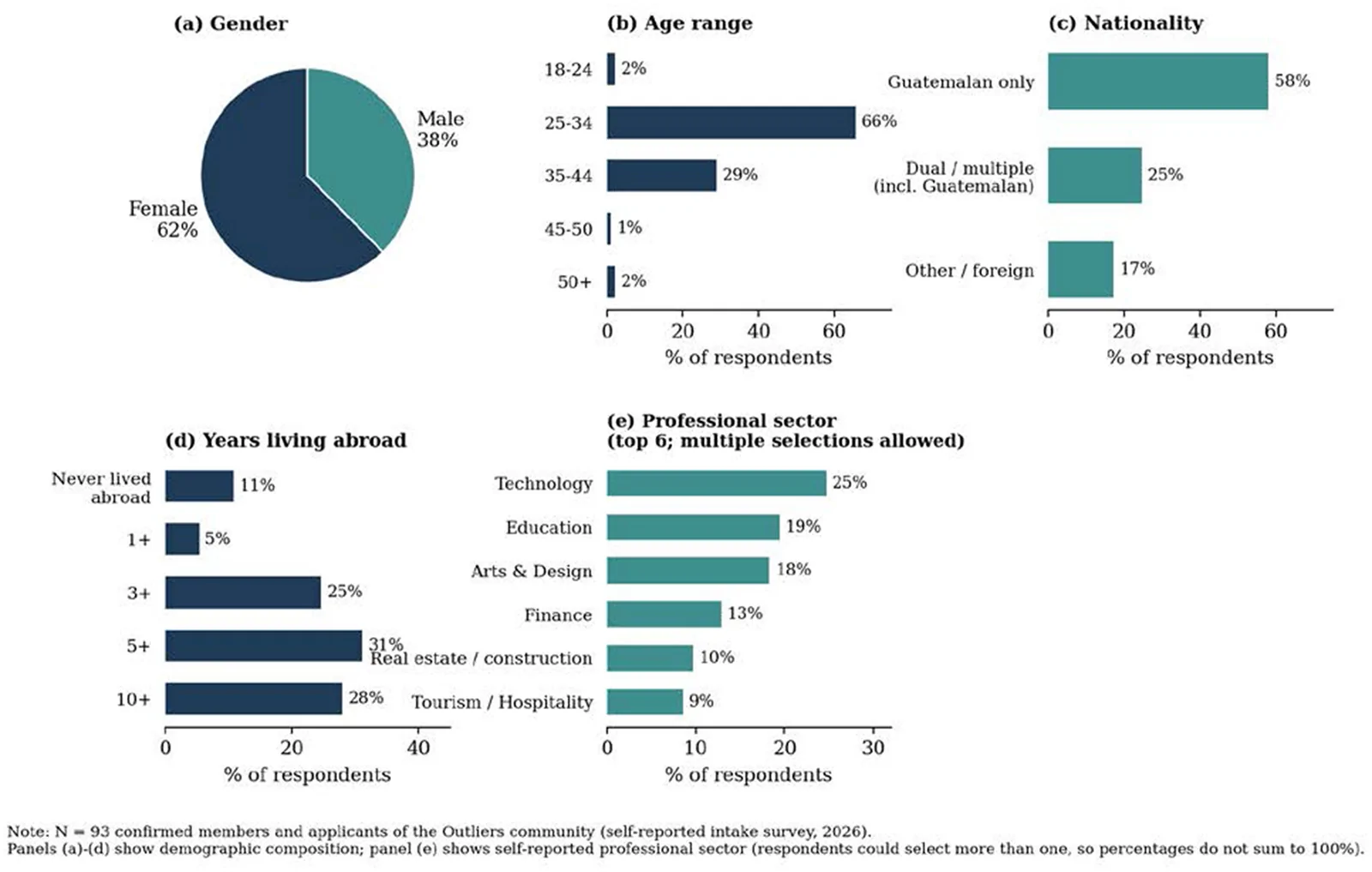
Demographic and professional profile of outliers members. *N* = 93 confirmed members and applicants (self-reported intake survey, 2026). **(a)**–**(d)**: gender, age range, nationality, and years living abroad. **(e)**: self-reported professional sector (multiple selections allowed; percentages do not sum to 100%).

The survey also highlighted a notable degree of sectoral diversity within the community, as shown in Figure 1, technology represented the largest professional category (25%), followed by education (19%), arts and design (18%), finance (13%), real estate (10%), and other sectors. From the perspective of innovation studies, this diversity is more than a descriptive characteristic, it constitutes a strategic asset for the community: it increases the likelihood of unexpected knowledge recombination across fields and reduces reliance on any single industry for the community's continued relevance and value creation. Hayek (1945) argued that knowledge is inherently dispersed across individuals and institutions, while subsequent research has shown that innovation frequently emerges from the recombination of ideas originating in different domains (Weitzman, 1998; Page, 2007). In the context of Guatemala, where professional communities frequently operate within sector-specific silos and opportunities for cross-sector interaction remain limited, the deliberate composition of Outliers creates opportunities for knowledge transfer across professional, institutional, and geographic boundaries. By bringing together internationally diaspora professionals, local entrepreneurs, and foreign residents within a single curated community, this creates conditions for the development of bridging social capital and interdisciplinary collaboration that might otherwise be difficult to achieve. For individual members, this translates into access to complementary expertise and collaboration opportunities beyond their own sector; for Guatemalan society more broadly, it creates a distributed channel through which internationally acquired knowledge can circulate across previously disconnected professional domains. This finding is consistent with research emphasizing the developmental value of transnational and cross-sector connections as mechanisms.

### 4.2 Emerging themes from survey responses

**Figure 2:**
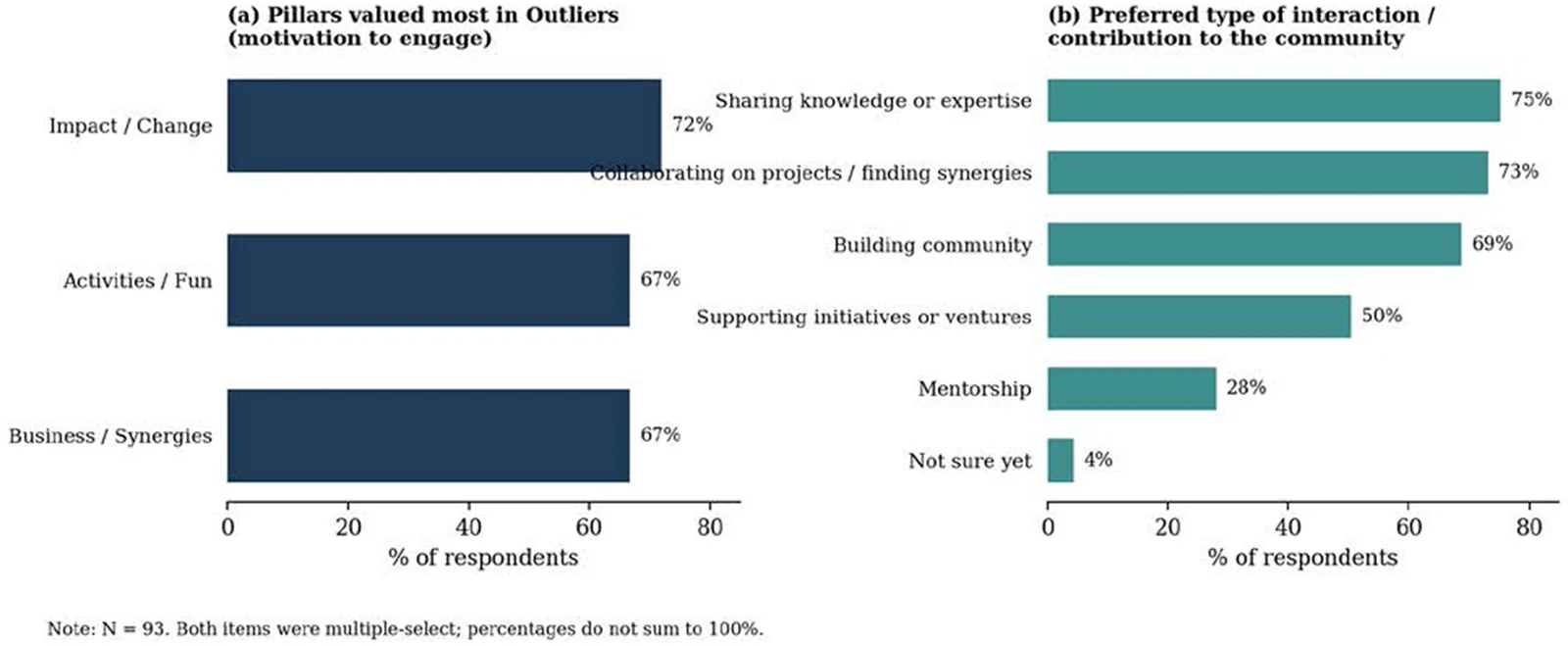
Motivation and interaction within the community. *N* = 93. (self-reported intake survey, 2026). **(a)**: pillars respondents value most in outliers. **(b)**: preferred types of contribution to the community. Both items were multiple-select.

To complement the quantitative survey results, open-ended survey responses were analyzed using an inductive thematic approach following the methodology proposed by Braun and Clarke (2006). Responses were systematically reviewed, coded and organized into broader analytical themes that captured shared perceptions among participants. This process provided additional insight into how members understood the community's purpose and value, while also offering qualitative corroboration of the patterns identified in the survey data. Four interconnected themes emerged consistently across responses. First, participants expressed a strong commitment to contributing to Guatemala's development (cited by 71% of respondents as a motivation for joining; see Figure 2). Second, respondents emphasized the importance of building meaningful connections that could facilitate access to new collaborators, opportunities, and specialized knowledge, consistent with the 82% of respondents who identified connecting professionals across sectors as Outliers's primary function. Third, many highlighted a willingness to share expertise, mentor peers, and engage in joint initiatives (75% expressed interest in sharing expertise and 73% in collaborating on projects). Finally, participants underscored the need for clearer structures and participation pathways to support sustained involvement in the community, echoing the 61% of respondents who cited limited time or unclear pathways as the primary barrier to more active participation.

The findings presented in Figure 2 illustrate the first theme: a strong purpose-driven orientation among community members. While meeting like-minded individuals was the most frequently cited motivation for joining Outliers (84%), a substantial majority of respondents also reported a desire to contribute to Guatemala's development (71%). Rather than representing separate motivations, these responses suggest that members do not view networking as an end itself, but rather as a mechanism for creating broader impact. This interpretation is reinforced by perceptions of the Outliers community's role. A large majority of respondents (82%) identified Outliers's primary function as connecting professionals across sectors, disciplines, and geographic boundaries. Rather than viewing it as a conventional event-based community, participants appear to value its ability to bring individuals with complementary expertise, shared interests, and common commitment to the country. In a context where Guatemala's innovation ecosystem remains fragmented and opportunities for meaningful cross-sector collaboration are often limited, these findings suggest that Outliers may serve as a mechanism for bridging professional divides among individual members, enabling skilled professionals to channel their expertise toward Guatemala's development goals; further research directly engaging institutional stakeholders would be needed to assess the extent to which institutional and individual professional goals are aligned.

This interpretation is reinforced by the profile of returnee members. Among 68 returnees surveyed, 69% reported that their decision to return was primarily driven by love for Guatemala and a desire to contribute to positive change, substantially outweighing more instrumental considerations such as employment opportunities (21%) or family business interests (25%). Even family-related motivations (41%) reflect a broader sense of place attachment, rooted in identity, belonging, and long-term commitment to the country (Tuan, 1977; Levitt and Glick Schiller, 2004). These findings suggest that many participants maintain strong emotional and social ties to Guatemala despite significant international exposure, creating favorable conditions for the mobilization of diaspora knowledge and experience toward collaborative initiatives with local entrepreneurs, institutions, and fellow diaspora professionals within the Outliers community.

The survey results further suggest that this commitment is accompanied by a substantial willingness to engage. A large majority expressed interest in sharing expertise (75%) or collaborating on projects (73%). Open-ended responses similarly emphasized a desire for purposeful interaction rather than passive networking. Participants consistently described the value of the community in terms of access to talented individuals, opportunities for collaboration, and the possibility of contributing to initiatives with tangible impact. From the perspective of diaspora knowledge network theory, these findings indicate the presence of significant latent human and social capital within the community (Brinkerhoff, 2008; Meyer and Brown, 1999a,b; Saxenian, 2006).

**Figure 3:**
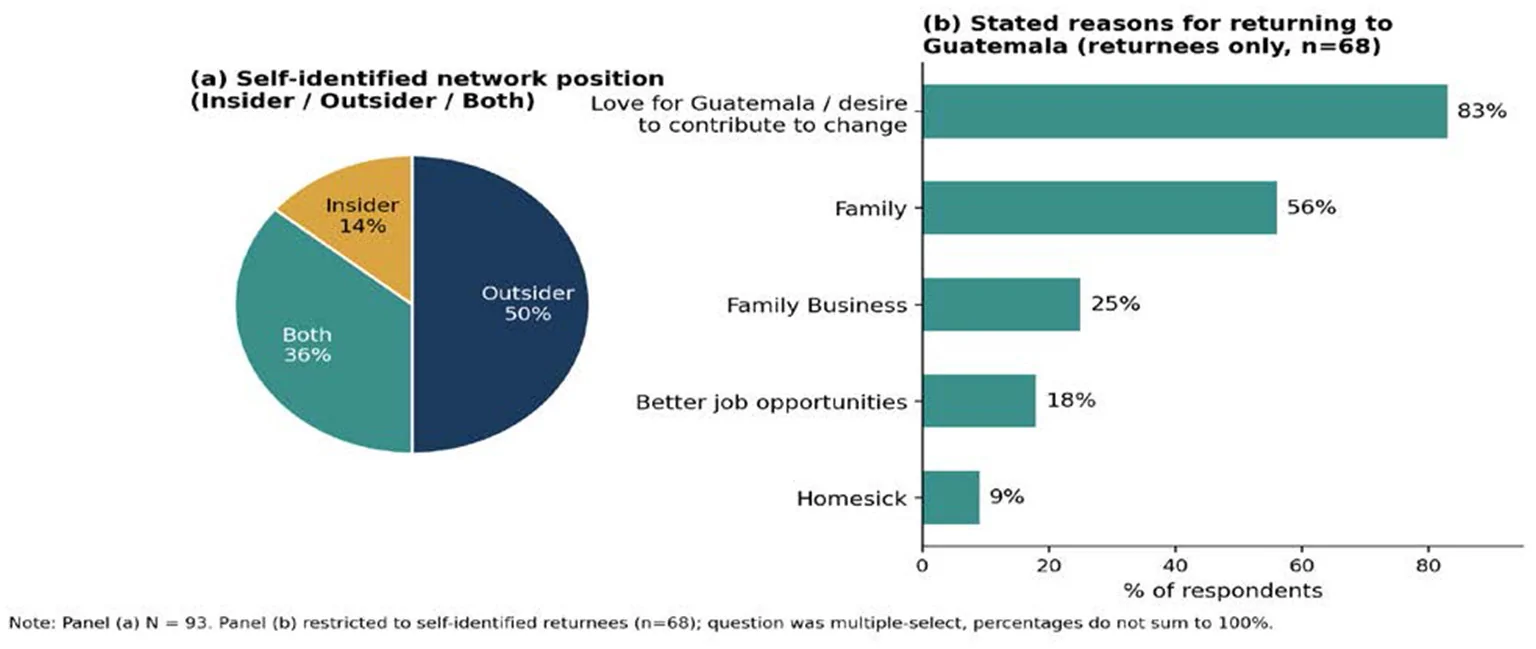
Identity and mobility trade-offs among members. **(a)**: self-identified position within the community (*N* = 93). **(b)**: stated reasons for returning to Guatemala among self-identified returnees (*n* = 68); multiple-select.

**Figure 4:**
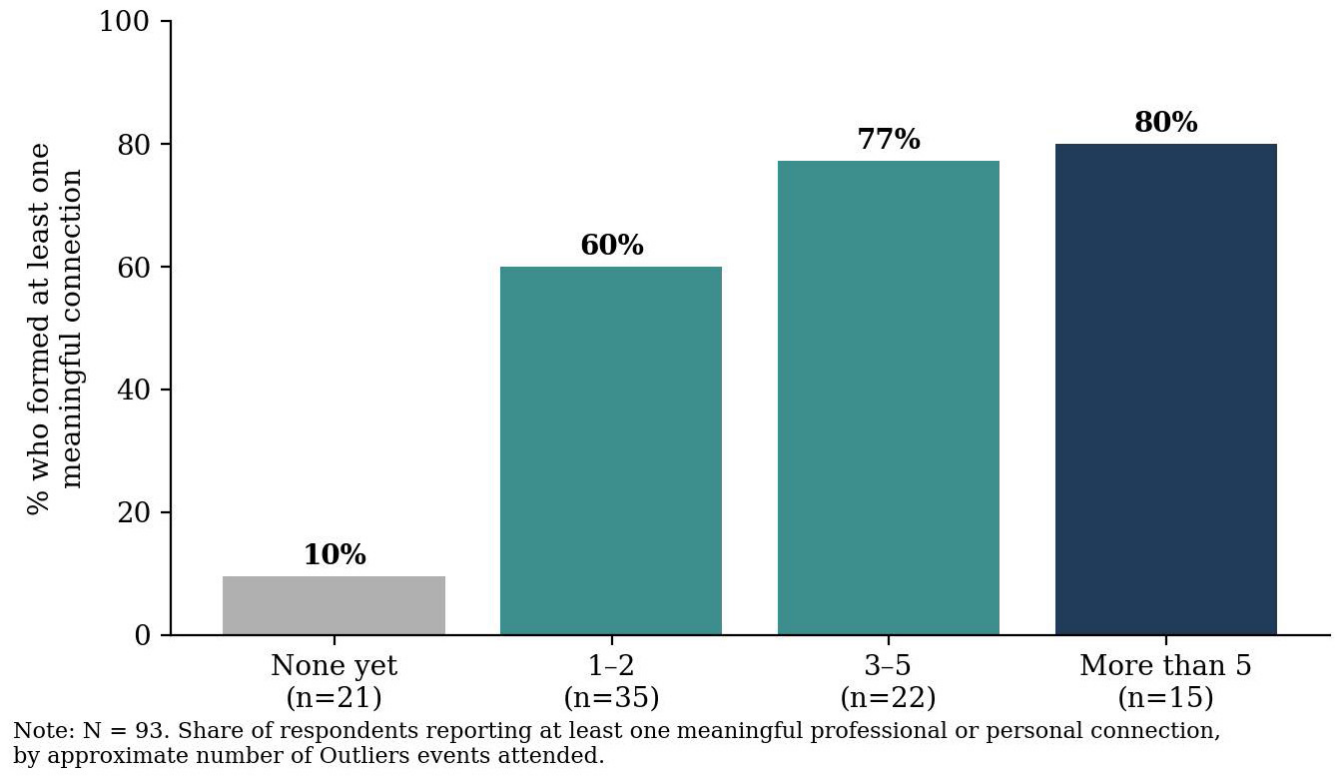
Event attendance and connection formation. *N* = 93. Share of respondents reporting at least one meaningful professional or personal connection, by approximate number of outliers events attended.

However, willingness alone does not automatically translate into collaboration. Figure 3 surfaces one of the most actionable findings in the dataset. Among members who had not yet attended a single Outliers event, only 10% reported forming a meaningful professional or personal connection through the community. That figure rose to 60% after just one or two events: a 50-point threshold effect that is neither gradual nor ambiguous. It is, in structural terms, an activation trigger: the moment of physical co-presence converts latent membership into relational reality. This pattern is consistent with what sociologists of community and network scholars have long argued, that weak ties require repeated, intentional interaction to consolidate into the kind of bridging social capital capable of generating collaborative opportunity (Granovetter, 1973; Putnam, 2000). In the Outliers context, events are not peripheral programming, they are the primary mechanism through which the community's dispersed human capital becomes accessible and actionable. Read alongside Figure 4, the logic is self-reinforcing: members who attend events form connections, and members who form connections demanded collaboration (38%) and introductions to relevant individuals (29%), precisely the outputs that cross-sector, globally shaped communities are uniquely positioned to deliver. For a community operating in a context where institutional trust is low and formal intermediaries are scarce, the event as a structured interaction space represents an unusually high-leverage intervention, one that transforms a directory of professionals into a living community with genuine productive potential (Brinkerhoff, 2008; Meyer and Brown, 1999a,b).

Yet the study also reveals an important implementation challenge. While willingness to collaborate was high, sustained engagement was constrained by time limitations and coordination challenges, with 61% of respondents citing limited time or scheduling as the primary barrier to more active participation. Others pointed to unclear pathways for involvement and limited coordination mechanisms. These findings suggest that the principal challenge facing Outliers is not attracting motivated members but creating organizational structures capable of channeling existing motivation into sustained engagement. As the community expanded, relational density appeared to grow faster than governance capacity. During the community's first year of operation, mechanisms such as thematic working groups, structured onboarding processes, and interest-based communities had not yet been systematically implemented. Their subsequent development reflects an important lesson emerging from the findings: while trust and connectivity provide the foundation for collaboration, they are insufficient on their own. Networks also require governance structures that reduce coordination costs, clarify opportunities for participation, and facilitate the translation of relationships into collective outcomes. In this sense, the experience of Outliers suggests that governance should not be understood as a bureaucratic layer imposed on a community, but as an enabling mechanism that allows social capital to be mobilized effectively. Governance does not need to be highly formalized, but some degree of organizational structure appears necessary to sustain engagement and convert goodwill into concrete collaboration. These findings also point to an underexplored transactional dimension of the community: members appear more willing to invest time when they perceive concrete reciprocal benefits, such as introductions or access to expertise, suggesting that transactional exchange, alongside relational trust, may function as a motivational and governance feature warranting further attention in future editions of Outliers's programming.

More broadly, the findings suggest that Outliers occupies a distinctive niche within Guatemala's professional ecosystem. Respondents frequently described the community as providing value not available through sector-specific associations, alumni networks, or traditional professional organizations. Its distinctive contribution lies in bringing together globally dispersed professionals, local entrepreneurs, and foreign residents committed to Guatemala's development within a single cross-sector community. In a context where actors across academia, the private sector, civil society, and the diaspora often operate in relative isolation, this combination of transnational perspectives and local commitment creates opportunities for connection and knowledge exchange that are unlikely to emerge through existing networks alone.

Taken together, the Outliers experience illustrates how intentionally designed networks can begin to create relational infrastructure within globally dispersed professional communities. While longer-term structural outcomes- such as formal collaborations, joint ventures, or partnerships—remain subjects for future research, the findings demonstrate that diaspora engagement is not solely a matter of attracting talent back to the country. Rather, it involves creating the relational and organizational conditions through which dispersed human capital can be connected, activated, and mobilized toward shared developmental objectives. In this sense, Outliers offers an early example of how diaspora knowledge networks can contribute to strengthening domestic innovation ecosystems in emerging economies. Although the community remains in an early stage of development, the findings suggest that intentionally designed communities can help bridge institutional, sectoral, and geographic divides while creating the connective capacity necessary for future collaboration. As such, the case may offer useful lessons for policymakers, universities, business associations, and other institutions seeking more structured approaches to diaspora engagement and ecosystem development.

## 5 Acknowledgments of conceptual and methodological constraints

This study is subject to some conceptual and methodological constraints that should be acknowledged. First, the research focuses on documenting the first year of the Outliers community, which limits the ability to assess long-term outcomes or sustained impacts on innovation capacity in Guatemala. As a result, the findings should be interpreted as preliminary insights rather than definitive conclusions about the long-term effectiveness of diaspora-driven networks.

Second, the study relies primarily on survey responses from members of the Outliers community. While the survey provides valuable insights into participants'motivations, perceptions, and experiences, the data are based on self-reported information, which may introduce response bias. Additionally, participation in both the community and the survey was voluntary, which may lead to self-selection bias, as individuals already interested in networking or innovation may have been more likely to participate.

Finally, as a community-based case study, the findings are context-specific and may not be directly generalizable to other innovation ecosystems. Guatemala's particular institutional conditions, migration patterns, and ecosystem fragmentation shape both the formation of the Outliers community and the meanings members assign to it. Despite these limitations, the findings suggest that diaspora-centered communities may represent a promising mechanism for strengthening connections between global talent and local innovation ecosystems. Future research could explore how such networks evolve, whether they lead to concrete collaborations or entrepreneurial initiatives, and how similar models might be adapted in other contexts seeking to leverage diaspora engagement for innovation and development.

## Data Availability

The original contributions presented in the study are included in the article/Supplementary material, further inquiries can be directed to the corresponding author/s.
