# Supplementary material for: Diaspora contributions: evidence from the Outliers network building in Guatemala
Source: Front Res Metr Anal. 2026 Aug 25;11:1852444. doi: 10.3389/frma.2026.1852444 (PMC13556154; doi:10.3389/frma.2026.1852444)
Supplement: Supplementary file 1 [file Supplementary_file_1.zip › Supplementary Material/Supplementary file.DOCX]

Annex II:

- Survey completed by members of the Outliers Community.

Link: <https://docs.google.com/forms/d/e/1FAIpQLSeOq529-cpAau0hBJnPZrZFtQKM5lXtWNK4DoA-_VYw4eZmmQ/viewform>
